# Supplementary figures and images for: Employing a systematic approach to biobanking and analyzing clinical and genetic data for advancing COVID-19 research
Source: Eur J Hum Genet. 2021 Jan 17;29(5):745–59. doi: 10.1038/s41431-020-00793-7 (PMC7811682; doi:10.1038/s41431-020-00793-7)

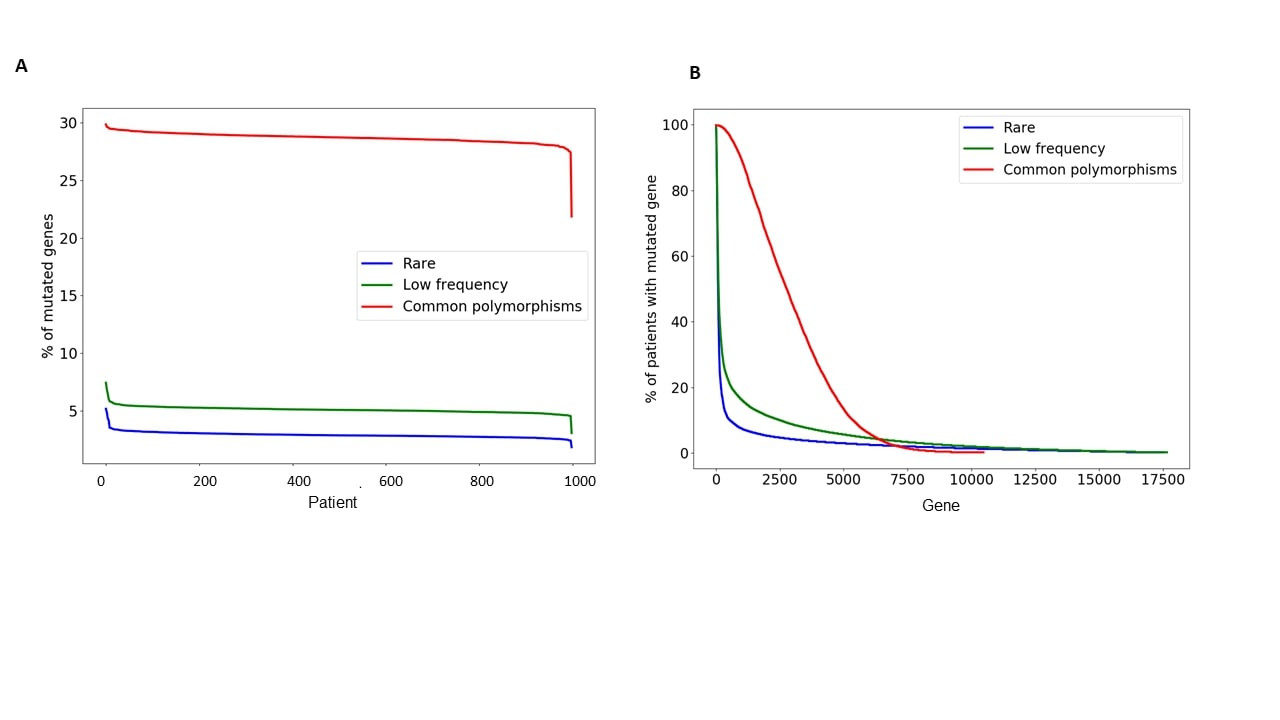

Supplement: Supplementary file 1 — Supplementary Figure 1 [file 41431_2020_793_MOESM1_ESM.jpg]
